# Construction and validation of a cuproptosis-related diagnostic gene signature for atrial fibrillation based on ensemble learning

**DOI:** 10.1186/s41065-023-00297-6

**Published:** 2023-08-24

**Authors:** Yixin Wang, Qiaozhu Wang, Peng Liu, Lingyan Jin, Xinghua Qin, Qiangsun Zheng

**Affiliations:** 1 Department of Cardiology, The Second Affiliated Hospital of Xi’an Jiaotong University, Xi’an, China; 2 Xi’an Key Laboratory of Special Medicine and Health Engineering, School of Life Sciences, Northwestern Polytechnical University, Xi’an, China

**Keywords:** Atrial fibrillation, Cuproptosis, Diagnostic signature, Bioinformatics, Ensemble learning

## Abstract

**Background:**

Atrial fibrillation (AF) is the most common type of cardiac arrhythmia. Nonetheless, the accurate diagnosis of this condition continues to pose a challenge when relying on conventional diagnostic techniques. Cell death is a key factor in the pathogenesis of AF. Existing investigations suggest that cuproptosis may also contribute to AF. This investigation aimed to identify a novel diagnostic gene signature associated with cuproptosis for AF using ensemble learning methods and discover the connection between AF and cuproptosis.

**Results:**

Two genes connected to cuproptosis, including solute carrier family 31 member 1 (SLC31A1) and lipoic acid synthetase (LIAS), were selected by integration of random forests and eXtreme Gradient Boosting algorithms. Subsequently, a diagnostic model was constructed that includes the two genes for AF using the Light Gradient Boosting Machine (LightGBM) algorithm with good performance (the area under the curve value > 0.75). The microRNA-transcription factor-messenger RNA network revealed that homeobox A9 (HOXA9) and Tet methylcytosine dioxygenase 1 (TET1) could target SLC31A1 and LIAS in AF. Functional enrichment analysis indicated that cuproptosis might be connected to immunocyte activities. Immunocyte infiltration analysis using the CIBERSORT algorithm suggested a greater level of neutrophils in the AF group. According to the outcomes of Spearman’s rank correlation analysis, there was a negative relation between SLC31A1 and resting dendritic cells and eosinophils. The study found a positive relationship between LIAS and eosinophils along with resting memory CD4^+^ T cells. Conversely, a negative correlation was detected between LIAS and CD8^+^ T cells and regulatory T cells.

**Conclusions:**

This study successfully constructed a cuproptosis-related diagnostic model for AF based on the LightGBM algorithm and validated its diagnostic efficacy. Cuproptosis may be regulated by HOXA9 and TET1 in AF. Cuproptosis might interact with infiltrating immunocytes in AF.

**Supplementary Information:**

The online version contains supplementary material available at 10.1186/s41065-023-00297-6.

## Background

Atrial fibrillation (AF) is a common cardiac arrhythmia in healthcare facilities, with a global prevalence exceeding 43 million individuals [1]. AF is a substantial risk factor for ischemic stroke, as it increases the probability of stroke by five times and is responsible for approximately one-third of all strokes [2–4]. Furthermore, strokes among subjects with AF are connected with elevated mortality compared to strokes in individuals without AF [5]. It is known that the administration of oral anticoagulation (OAC) could significantly mitigate the risk of AF-related stroke [6, 7]. However, since AF can escape traditional monitoring techniques due to its often asymptomatic and paroxysmal nature [8], leading to delayed onset of OAC, ischemic stroke is often the initial sign of AF [9]. Therefore, novel diagnostic approaches supplementing the current methods for the timely detection of AF are urgently required.

The pathophysiological pathways for the beginning and perpetuation of AF are extremely complex. There is a growing body of evidence suggesting that genetic factors are a significant contributor to the development of AF. Genome-wide association studies have identified approximately 140 genetic loci that are associated with AF [10]. Recently, the rapid advancement in microarray technology has enabled the identification of gene biomarkers associated with AF [11, 12], enabling the development of new diagnostic models based on genes for diagnosing AF. Additionally, electrical and structural remodeling, the predominant mechanism underlying AF, has been associated with various types of cell death, such as ferroptosis, necroptosis, apoptosis, and autophagy [13–16]. Recently, Tsvetkov et al. introduced a new form of cellular death known as cuproptosis, which is triggered by excessive accumulation of copper (Cu) [17]. Grandis et al. reported that Wilson’s disease (WD), a disease resulting from abnormal Cu metabolism, induced a higher risk of AF [18], which may be a consequence of myocardial Cu deposition [19]. Significantly, Cu is involved in immunity [20].

The immune response and inflammation are two crucial mechanisms in AF pathogenesis. Numerous inflammatory biomarkers, including interleukins, C-reactive protein, and tumor necrosis factor-α, have been associated with AF [21]. Relevant studies revealed a vital role of the immunocyte infiltration of atrium in the pathogenesis of AF [22]. In summary, it is reasonable to consider that cuproptosis is tightly connected to the pathogenesis of AF. Therefore, the establishment of a gene signature linked to cuproptosis may provide the foundation to further investigate the association between AF and cuproptosis, which can shed new light on the diagnosis and management of individuals with AF.

Machine learning (ML), involving procedures that learn to make decisions from data, demonstrated success and scalability in the diagnosis and prognosis of AF [23]. Ensemble learning (EL) is a subfield of ML. The utilization of EL algorithms in computational biology has become more prevalent as a result of their distinct benefits in managing limited sample sizes, intricate data constructions, and high-dimensional data [24]. To our knowledge, the application of EL algorithms such as Random Forests (RF) [25], eXtreme Gradient Boosting (XGBoost) [26], and Light Gradient Boosting Machine (LightGBM) [27] has not yet been reported for the diagnostic gene signature of AF. RF algorithm allows predictors to be ranked according to their importance in a regression or classification problem [28]. XGBoost and LightGBM algorithms are both based on gradient-boosting tree-based methods. XGBoost could analyze feature importance internally throughout the learning process and provide scores for all features. The LightGBM algorithm exhibits superior performance compared to XGBoost, with notable enhancements in performance, training speed, and accuracy [29].

**Fig. 1:**
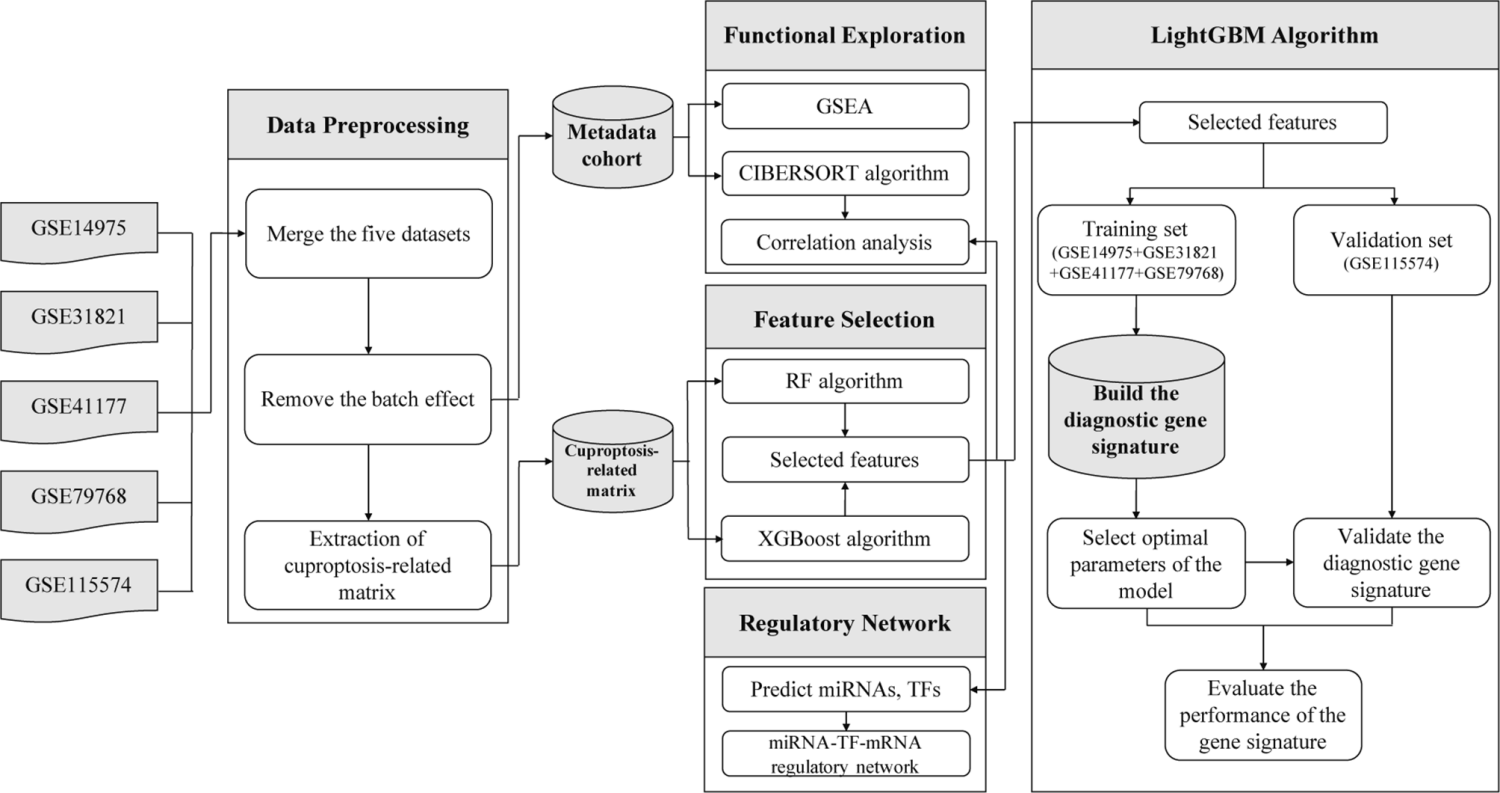
Flowchart of this study

This study aims to discover the association between cuproptosis-associated genes and AF, investigate the diagnostic importance of cuproptosis-associated gene signature based on the LightGBM algorithm, study the correlations between cuproptosis and immunocyte infiltration, and construct the microRNA (miRNA)-transcription factor (TF)-messenger RNA (mRNA) regulatory network of the genes. The analysis process of this investigation is illustrated below (Fig. 1).

## Materials and methods

### Acquisition and preprocessing of datasets

This study employed “atrial fibrillation” as the designated keyword, specified the study organism as “Homo Sapiens”, and identified the study type as “Expression profiling by array”. Subsequently, it conducted a thorough exploration of the atrial fibrillation-associated datasets within the Gene Expression Omnibus (GEO) database (https://www.ncbi.nlm.nih.gov/geo/). Finally, the matrix of expression for five unique datasets, namely GSE79768, GSE31821, GSE41177, GSE14975, and GSE115574 (Table 1), was obtained from the GEO repository. Samples of atrial tissue from sinus rhythm (SR) controls and patients with AF were selected for analysis. The Affymetrix Human Genome U133 Plus 2.0 Array (GPL570) was utilized to annotate all five datasets. The conversion of probes in each dataset was performed utilizing annotation files with ActivePerl (5.18.4) to obtain corresponding gene symbols. GSE14975 was first transformed into log2 transformed first to ensure consistency with the other four datasets, which had been preprocessed with log2 transformation before. In order to combine these five datasets as a metadata cohort, the batch effect should be removed. Batch normalization was performed for the merged expression data of five datasets in R (4.1.0) with the “sva” package(3.40.0) [30], and the ComBat method was used to normalize the expression values from different datasets [31]. **Table**S1 presents that 13 cuproptosis-related genes were collected from prior research [17]. Cuproptosis-related gene matrix was extracted from the metadata cohort based on the cuproptosis-related genes using R (4.1.0).

**Table 1:** Details of the five datasets

Series accession	Experiment type	Sample size (selected/total)	Sample size (AF/SR)	Contributors	Country	Last update date
GSE14975	Expression profiling by array	10/10	5/5	Adam O, et al.	Germany	2019/3/25
GSE31821	Expression profiling by array	5/6	3/2	Morel E, et al.	France	2019/3/25
GSE41177	Expression profiling by array	38/38	32/6	Yeh Y, et al.	China (Taiwan)	2019/3/25
GSE79768	Expression profiling by array	26/26	14/12	Tsai FC, et al.	China (Taiwan)	2019/3/25
GSE115574	Expression profiling by array	59/59	28/31	Deniz GC, et al.	Turkey	2021/12/13

### Cuproptosis-related gene selection utilizing RF and XGBoost algorithms

To identify cuproptosis-related diagnostic variables tightly related to AF, RF, and XGBoost, algorithms were implemented in the cuproptosis-related matrix. RF algorithm was conducted to compute the importance score with 1000 classification trees constructed initially using the “randomForest” package (4.6–14) [28]. Subsequently, the optimal number of trees to grow (ntree) was determined according to the minimum error rate. The features with Gini importance ranked as the top 3 were considered. XGBoost algorithm was implemented with parameters set as “the learning rate (eta) = 0.3, maximum depth of a tree (max_depth) = 6, max number of boosting iterations (nrounds) = 10” through the “xgboost” package (1.5.0.2, https://github.com/dmlc/xgboost). The features with relative Gain-importance ranking top 3 were selected. Finally, the overlapping cuproptosis-related genes from the two algorithms were selected to establish the diagnostic gene signature, and the Venn diagram was produced utilizing VENNY 2.1 (https://bioinfogp.cnb.csic.es/tools/venny/).

### The diagnostic gene signature construction and validation

The present study artificially split the cuproptosis-related matrix into two sets: the training set containing GSE14975, GSE31821, GSE41177, and GSE79768, and the validation set containing GSE115574. The cuproptosis-related genes selected by RF and XGBoost algorithms were submitted to the LightGBM algorithm to build the diagnostic gene signature in training set utilizing the “lightgbm” package (3.3.2, https://github.com/Microsoft/LightGBM). The optimal parameters of a LightGBM-based model, including minimal sum Hessian in one leaf (min_sum_hessian_in_leaf), L1 regularization (lambda_l1), L2 regularization (lambda_l2), and the ratio of structures randomly chosen on every iteration (feature_fraction), were determined based on the minimum square loss during the process of training. Finally, the diagnostic gene signature was built with the optimal parameters and other parameters set as “eta = 0.1, nrounds = 100” and validated in the validation set.

### Evaluation of the LightGBM-based Diagnostic Gene signature

The efficiency of the diagnostic gene signature according to LightGBM was assessed utilizing the area under the curve (AUC) of receiver operator characteristic (ROC) and precision-recall (PR) curves. Specifically, the ROC-AUC and PR-AUC were utilized for this purpose. ROC curves were generated by the “pROC” package (1.18.0) [32], while PR curves were developed through the “ggplot2” package [33]. In general, an AUC value > 0.75 was used as a threshold for good discriminating capacity.

### Functional Enrichment Analysis through Gene Set Enrichment Analysis (GSEA)

In the metadata cohort, GSEA [34] was implemented in the metadata cohort through the “clusterProfiler” package (4.0.5) [35] to discover the functional divergence between AF and SR. Gene ontology (GO)-biological progress (BP), Kyoto Encyclopedia of Genes and Genomes (KEGG), and Hallmark enrichment analysis were performed, respectively. The reference gene sets “h.all.v7.5.1.symbols.gmt”, “c2.cp.kegg.v7.5.1.symbols.gmt”, and “c5.go.v7.5.1.symbols.gmt” were obtained from Molecular Signatures Database (MSigDB, https://www.gsea-msigdb.org/gsea/msigdb/index.jsp). The significance threshold was established as an adjusted P-value (adj.p) of less than 0.05 and a false discovery rate (FDR) of less than 0.25.

### Interactions between cuproptosis-related genes and immunocyte infiltration

The CIBERSORT algorithm [36] was performed to detect the relative proportions of 22 forms of infiltrating immunocytes (LM22) in patients with AF. The CIBERSORT algorithm was run using the LM22 gene set at 1000 permutations. The *p* < 0.05 served as the criteria for the inclusion of samples. The Wilcox assessment was adopted to compare the variations in the proportions of immunocytes among AF patients and SR controls. A statistical significance level of p < 0.05 was considered acceptable. Correlation analysis between the cuproptosis-associated genes selected by EL algorithms and Spearman’s rank correlation analysis was utilized to conduct an immune response. Given that the |correlation coefficient (*R*)| < 0.2 indicates no correlation [37], it is necessary to set the criterion for the significance of correlation analysis as |*R*| $\ge$ 0.2 and *p* < 0.05.

### Construction of MicroRNA (miRNA)-Transcription factor (TF)-messenger RNA(mRNA) network

The genes associated with Cuproptosis, as identified by two EL algorithms, were subjected to analysis utilizing version 1.14.0 of the “multiMiR” package [38]. This analysis aimed to identify miRNAs in verified miRNA-target databases (miRecords, miRTarBase, and TarBase) as well as anticipated miRNA-target databases (DIANA-microT, ElMMo, MicroCosm, miRanda, miRDB, PicTar, PITA, and TargetScan), respectively. To improve the accuracy of prediction, only miRNAs fished by at least three predicted or two verified databases were retained. Meanwhile, TFs targeting cuproptosis-related genes were predicted using the “TF perturbations followed by expression table” module in Enrichr (https://maayanlab.cloud/Enrichr/) [39]. The *adj.p* < 0.05 served as a criterion for the inclusion of TFs, and the miRNA-TF-mRNA network was visualized and analyzed by the Cytoscape program (3.8.2) after identifying the modulatory relationships of miRNA-TF-mRNA [40].

### Statistical analysis

The statistical analyses were performed utilizing R (version 4.1.0). A significant difference is typically denoted by p < 0.05.

## Results

### Batch normalization of data

**Fig. 2:**
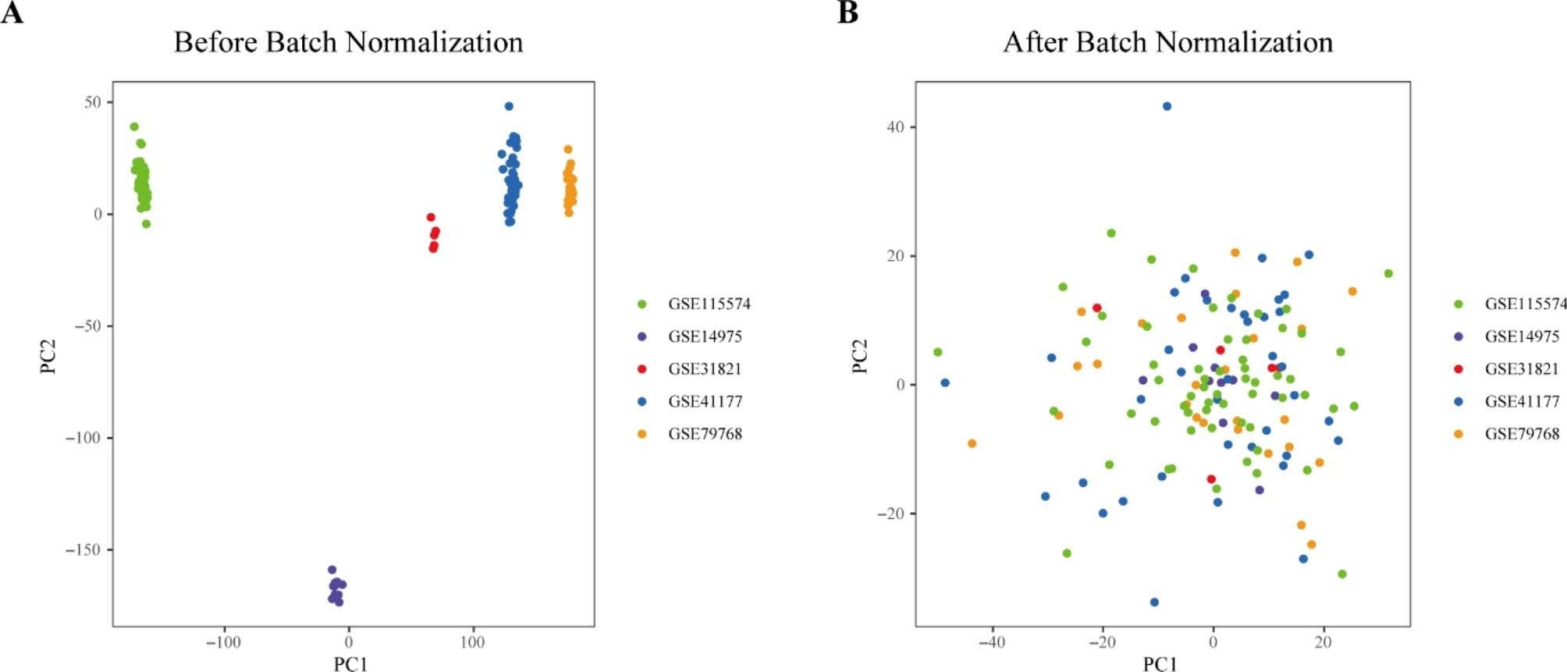
Scatter-plot of PCA. (**A**) Before batch normalization. (**B**) After batch normalization

In the analysis of five datasets, principal component analysis (PCA) was applied to investigate the sample clustering patterns before and after batch normalization. Before the batch normalization, the specimens were collected in batches depending on the top two principal components (PCs, Fig. 2A). Conversely, the scatter plot of normalized data suggested that the batch effect was successfully removed(Fig. 2B).

### Progression of the cuproptosis-associated diagnostic gene signature

**Fig. 3:**
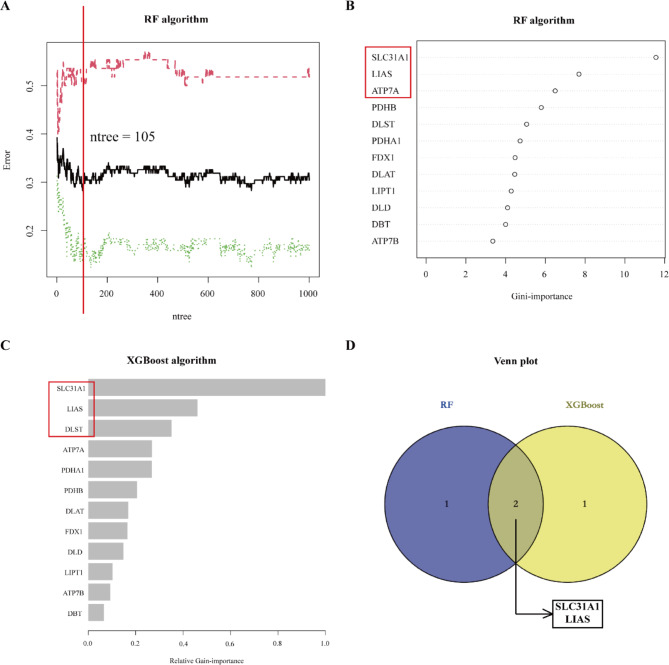
Feature selection with RF and XGBoost algorithms. (**A**) Relationship between the error rate and the number of classification trees. The error rate is minimum when ntree = 105. (**B**) Gini-importance of the 12 cuproptosis-related genes. (**C**) Relative Gain-importance of the 12 cuproptosis-related genes. (**D**) Venn plot demonstrating two features shared by RF and XGBoost algorithms

The cuproptosis-related matrix contained 12 cuproptosis-related genes since glycine cleavage system protein H (GCSH) did not exist in the metadata cohort. RF algorithm identified three features, including lipoic acid synthetase (LIAS), ATPase copper transporting Alpha (ATP7A), and solute carrier family 31 member 1 (SLC31A1) (Fig. 3A–B). Meanwhile, XGBoost selected three cuproptosis-related genes, including SLC31A1, LIAS, and dihydrolipoamide s-succinyltransferase (DLST, Fig. 3C). The two overlapping features (SLC31A1, LIAS), which were tightly related to AF, were ultimately selected to build the diagnostic gene signature (Fig. 3D). During the model training procedure, the optimal parameters of LightGBM-based model were finally determined as “lambda_l1 = 0, lambda_l2 = 1, min_sum_hessian_in_leaf = 0, feature_fraction = 0.8”. The diagnostic gene signature with optimal parameters was validated in the validation set.

### Diagnostic efficacy of the cuproptosis-related diagnostic gene signature

**Fig. 4:**
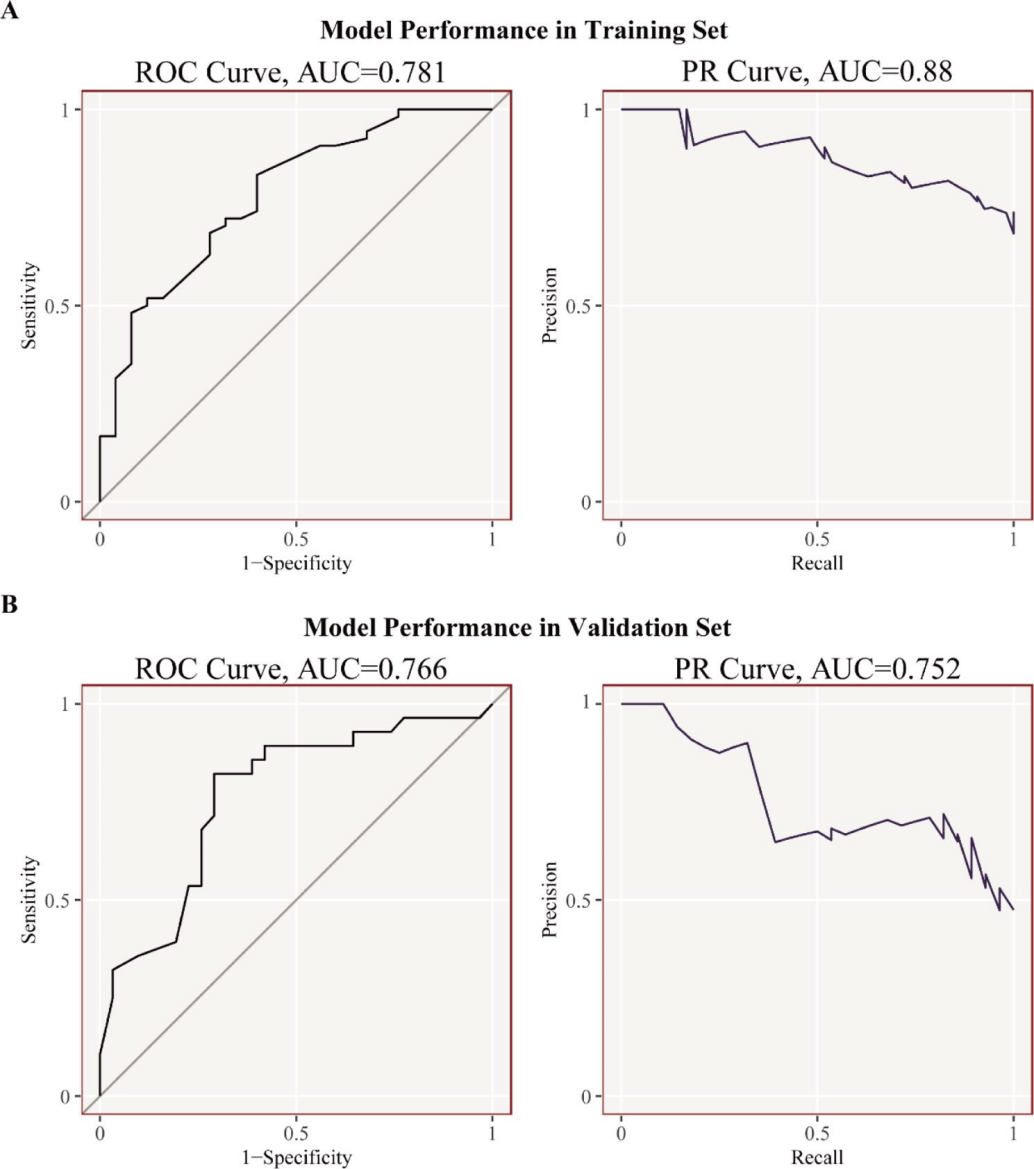
PR and ROC curves of the diagnostic gene signature. (**A**) Training set. (**B**) Validation set

AUC-ROCs only compare the true- and false-positive rates, which means that AUC-ROCs only depict the capability of signature to discriminate between AF and SR. However, the signature is expected to have better performance in identifying AF but not SR in clinical scenarios. Consequently, PR curves comparing true and predicted positives were employed to assess the signature performance. The value of ROC-AUCs and PR-AUCs in both training and validation sets was higher than 0.75 (Fig. 4A–B), indicating that the signature had a good utility for discriminating between AF and SR, and performed a good separation that specifically mapped to AF.

### Functional enrichment analysis

**Fig. 5:**
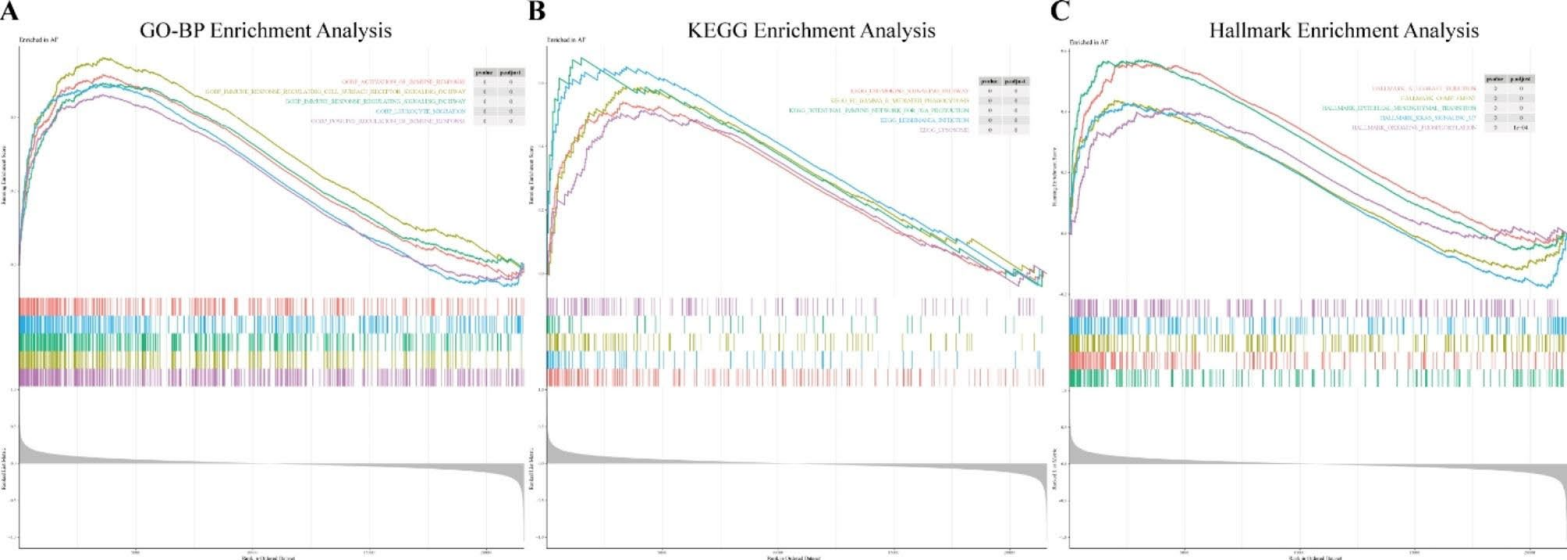
Functional analysis by GSEA. (**A**) Top 5 GO functions. (**B**) Top 5 KEGG pathways. (**C**) Top 5 Hallmark terms

To comprehensively understand the variations in gene roles and mechanisms between groups characterized by the cuproptosis-related diagnostic gene signature, GSEA was conducted. Since the cuproptosis-related diagnostic gene signature displayed a good separation specifically mapped to AF, the functional annotations enriched in the AF group were valued. In GO-BP enrichment analysis, BP terms were significantly enriched in the immune response, including activation of the immune response, immune response-regulating signaling mechanism, immune response-regulating cell surface receptor signaling pathway, positive regulation of immune response, and leukocyte migration (Fig. 5A). KEGG enrichment analysis revealed that remarkably enriched pathways in AF were mainly immunocyte-related, such as Fc gamma receptor (Fc gamma R)-mediated phagocytosis, chemokine signaling mechanism, intestinal immune network for immunoglobulin A (IgA) production, Leishmania infection and lysosome (Fig. 5B). Meanwhile, Hallmark terms significantly enriched in AF were allograft rejection and complement, which were tightly related to immunity (Fig. 5C).

In summary, the findings demonstrate that the cuproptosis-related diagnostic signature may be tightly related to the biological activities of immunocytes, which has an indispensable function in AF pathogenesis.

### Immunocyte infiltration and correlation analysis

**Fig. 6:**
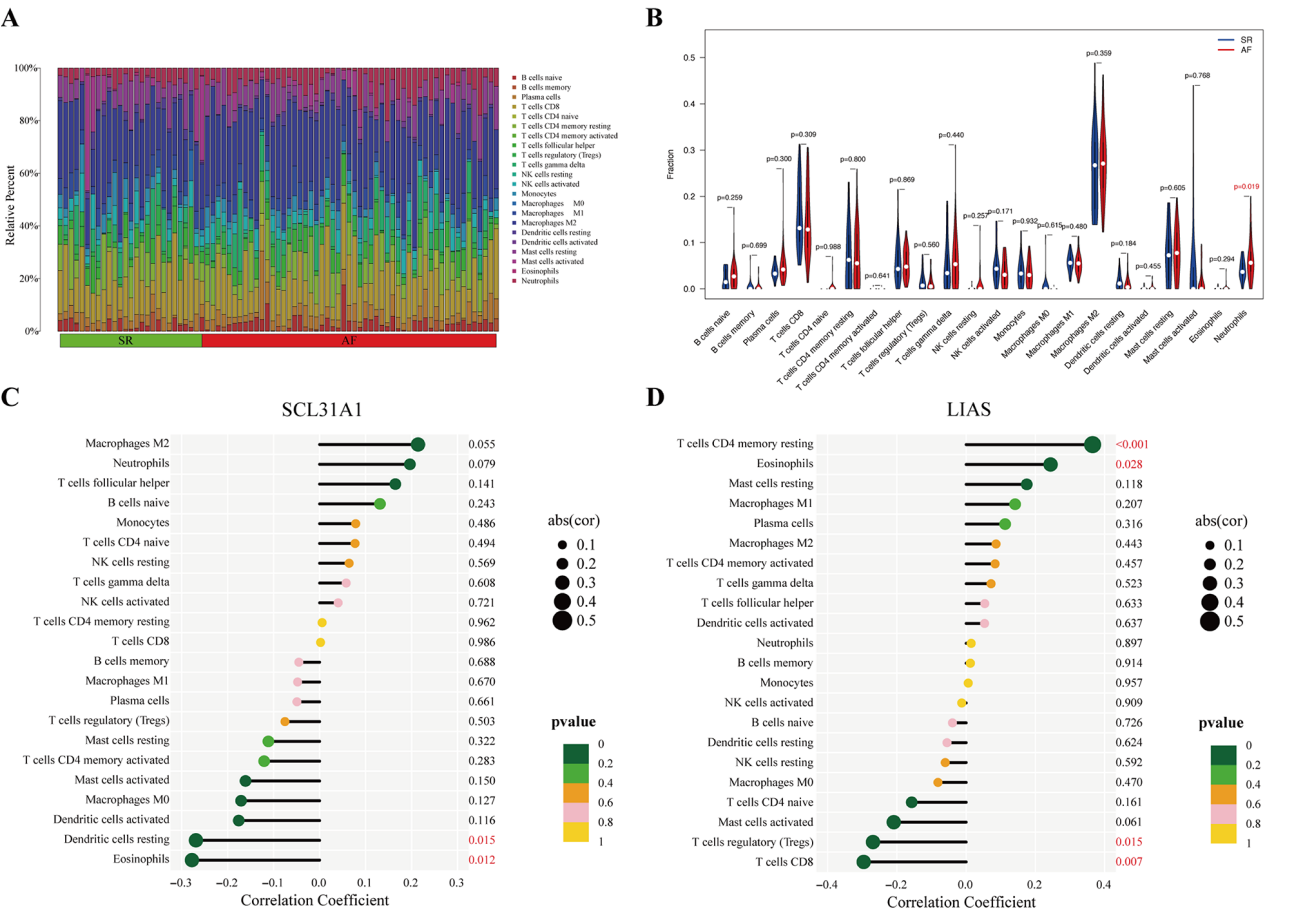
Immunocyte infiltration and correlation analysis. (**A**) Bar plot displaying the composition of 22 forms of immunocytes between AF and SR samples displayed by different colors. (**B**) Grouped violin plot comparing 22 types of immunocytes between AF patients and SR controls. (**C**) Correlation between SLC31A1 and 22 types of immunocytes. (**D**) Correlation between LIAS and 22 types of immunocytes

**Fig. 7:**
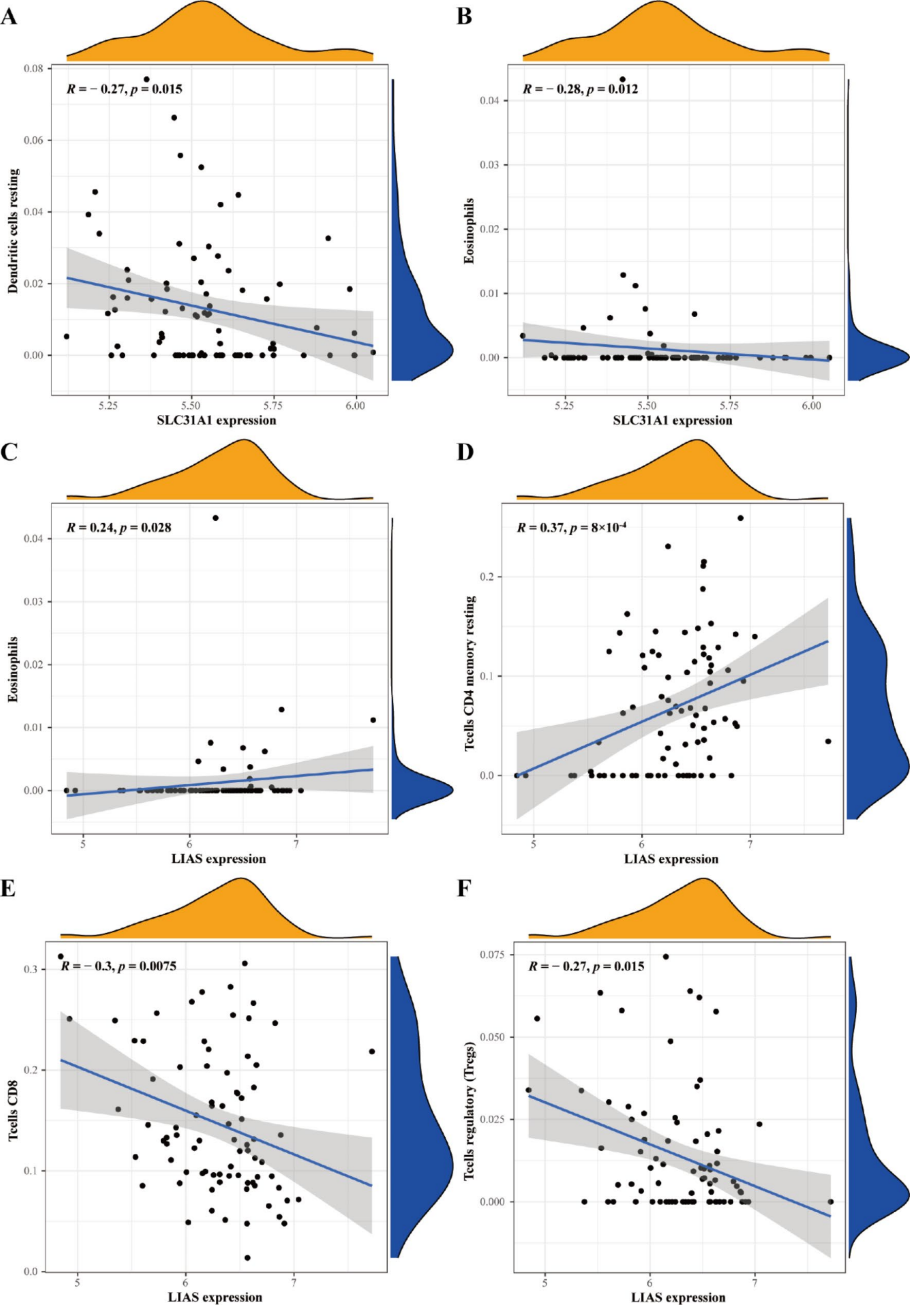
Correlation analysis of the two genes associated with Cuproptosis and their corresponding infiltrating immunocytes. (**A–B**) SCL31A1. (**C–F**) LIAS

Depending on the functional enrichment analysis outcomes, the CIBERSORT procedure was employed to quantify the composition of 22 forms of immunocytes between the AF and SR groups categorized by the cuproptosis-related diagnostic gene signature. The outcomes exhibited that neutrophil infiltration was significantly elevated in AF patients (Fig. 6A–B). Moreover, correlation analysis was conducted between the two cuproptosis-related genes belonging to the diagnostic signature and infiltrating immunocytes. SLC31A1 was negatively associated with resting dendritic cells (*R* = -0.27, *p* = 0.015) and eosinophils (*R* = -0.28, *p* = 0.012, Fig. 6 C and 7 A–B). LIAS was positively correlated with eosinophils (*R* = 0.24, *p* = 0.028) and resting memory CD4^+^ T cells (*R* = 0.37, *p* = 8$\times$10^−4^), but negatively related to CD8^+^ T cells (*R* = -0.3, *p* = 0.0075) and regulatory T cells (Tregs, *R* = -0.27, *p* = 0.015, Figs. 6D and 7 C–F).

### Regulatory Network of cuproptosis-related genes

**Fig. 8:**
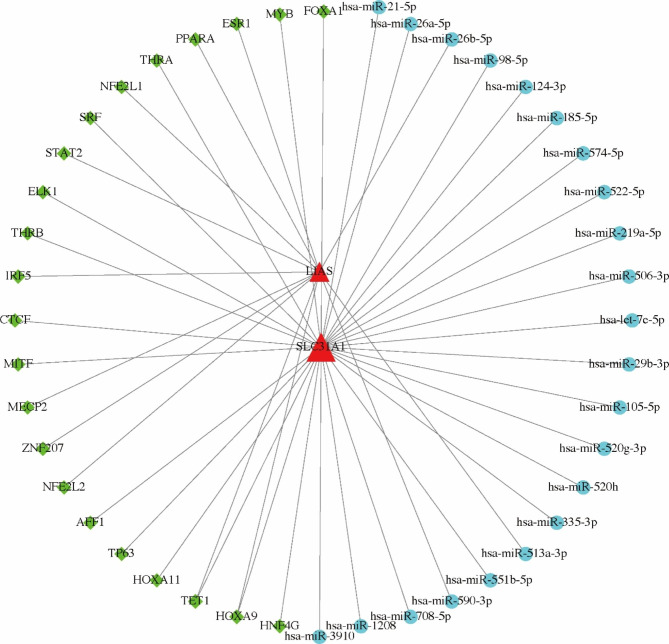
The miRNA-TF-mRNA network of cuproptosis-related genes. Blue circles represent miRNAs, green diamonds represent TFs, and red triangles represent mRNAs. The edges represent the relationship of miRNA-mRNA or TF-mRNA. The greater the degree of the node, the larger the node

The miRNA-TF-mRNA network containing 2 mRNAs, 22 miRNAs, and 22 TFs was constructed (Fig. 8). In the network, SLC31A1 mRNA and LIAS mRNA were targeted by homeobox A9 (HOXA9) and Tet methylcytosine dioxygenase 1 (TET1).

## Discussion

Multiple mechanisms, including genetic factors, various types of cell death, immunocyte infiltration, and inflammation, are connected to the incidence and development of AF. Up to now, the diagnosis of AF has still been a challenge because it is often paroxysmal and asymptomatic in clinics. Gaining more insight into the underlying mechanism of AF would enable novel methods to diagnose AF. Tsvetkov et al. reported that the binding of Cu to lipoylated ingredients in the tricarboxylic acid cycle causes cuproptosis, a recently recognized type of cell death. [17]. Although previous studies found that not only Cu itself but also WD, a Cu toxicity disease, were related to AF [18, 41], there are still no studies available to suggest any explainable pathogenesis in detail of Cu triggering or maintaining AF. Thus, the purpose of this investigation is not only to identify a novel diagnostic gene signature that may be available to assist clinical diagnosis of AF but also to investigate the relationship between Cu and AF from the aspect of cuproptosis.

This investigation is the first to identify the diagnostic gene signature connected to cuproptosis through bioinformatics methods integrating with EL algorithms, such as RF, XGBoost, and LightGBM algorithms. A cuproptosis-related diagnostic gene signature featuring two genes (SLC31A1 and LIAS) was finally established and validated with good efficacy in identifying AF, specifically with the value of ROC-AUCs and PR-AUCs exceeding 0.75. In contrast, many medical investigations are further required to verify the diagnosis significance of cuproptosis-related diagnostic gene signature. SLC31A1, also known as “copper transporter 1 (CRT1)”, encodes the protein serving as an increased-affinity Cu importer in the cell membrane. Kim et al. stated that the cardiac-specific knockout of SLC31A1 resulted in morphological, histological, molecular, and physiological hallmarks of cardiomyopathy [42], indicating that SLC31A1 is responsible for preserving the typical cardiac structure and function. LIAS encodes an iron-sulfur enzyme located in the mitochondrion, catalyzing the biosynthesis of lipoic acid. Previous studies reported that alteration of LIAS gene expression affected the development of atherosclerosis [43–45], which is a chronic inflammatory disease and one of the risk factors for AF [46]. Therefore, SLC31A1 and LIAS are likely to impact the AF pathogenesis. Since the direct function of the two genes in AF has been little explored, further studies may focus on the underlying mechanism linking the two cuproptosis-related genes and AF.

In the miRNA-TF-mRNA regulatory network, HOXA9 and TET1 could simultaneously regulate SLC31A1 mRNA and LIAS mRNA. A new investigation by Cai et al. revealed that HOXA9, a member of the Homeobox gene family encoding several greatly conservative progressive transcription factors, could promote cardiomyocyte hypertrophy [47], which is one of the most important structural remodeling features in AF [48]. TET1 is a 5-methylcytosine hydroxylase that initiates the DNA demethylation process [49]. Zhou et al. informed that TET1 was related to the direct cardiac reprogramming of fibroblasts into cardiomyocytes in humans [50]. Since atrial fibrosis involving an abnormal proliferation of cardiac fibroblasts and loss of cardiomyocytes is a characteristic of structural remodeling in AF [51], TET1 can serve as a crucial regulatory point to attenuate structural remodeling by direct cardiac reprogramming in AF. In view of the potential association between the two TFs and AF, it seems reasonable to assume that HOXA9 and TET1 might target SLC31A1 mRNA and LIAS mRNA in the pathogenesis of AF by regulating cuproptosis. However, there is still no study focusing on the interactions between the two TFs and the two cuproptosis-related genes, so further studies will be needed.

The phenomenon of cuproptosis has not been extensively investigated in scientific research. The results of GSEA indicated that various immunocyte-related functions and mechanisms were significantly enriched in the AF group, which could be specifically identified through the cuproptosis-related gene signature with good performance. Therefore, it is a justifiable hypothesis that cuproptosis may influence the structure of immunocytes infiltrating the atria.

The proportion of neutrophils among individuals with AF was found to be more elevated in comparison to the SR control group. Neutrophils are the most abundant type of leukocyte and have been linked to the regulation of cardiovascular inflammation [52]. The neutrophil-to-lymphocyte ratio elevation is related to the incidence and recurrence of AF [53]. Furthermore, studies showed that neutrophils infiltrating the myocardial interstitium release myeloperoxidase and reactive oxygen species, which induce atrial fibrosis and fibrillation [54]. Furthermore, Babu et al. revealed that the function of neutrophils was sensitive to Cu status [55]. The present findings coincide with previous studies, not only confirming the accuracy of findings but also suggesting the complexity between cuproptosis and neutrophils in AF.

The study conducted a correlation analysis between two genes associated with cuproptosis and infiltrating immunocytes. The findings demonstrated that SLC31A1 exhibited a negative relationship with eosinophils and resting dendritic cells. The study found a positive correlation between LIAS and eosinophils in addition to resting memory CD4^+^ T cells, while a negative relationship was detected between LIAS and CD8^+^ T cells and Tregs. A previous study by Tian et al. indicated that LIAS overexpression led to a reduction in CD4 + T cell infiltration and an increase in Treg number in peripheral blood in atherosclerosis [45], but similar investigations are not carried out in the case of AF. Given the lack of research, the sophisticated interactions between cuproptosis-related genes and immunocytes should be investigated in depth on the basis of the assumption mentioned previously.

It is crucial to take into account the restrictions of this investigation while interpreting the outcomes. First, the findings were derived exclusively from public databases using bioinformatics methods. Even though the present results were validated with a validation set, further clinical investigations with large sample sizes are essential for fully evaluating the feasibility of results. Second, this investigation represents the initial attempt to elucidate the correlation between cuproptosis and AF. Currently, research focusing on cuproptosis is still scarce. More in vivo or in vitro functional experiments are required to explore the underlying pathways that link cuproptosis to AF based on the results of this investigation. Third, a bioinformatics approach was used in this study to examine miRNA-TF-mRNA triple interactions associated with cuproptosis-related AF development. Nevertheless, the obtained miRNA-TF-mRNA interactions still require further experimental verification.

## Conclusion

In summary, this investigation demonstrated that cuproptosis was closely related to AF. A 2-gene diagnostic signature that includes cuproptosis-related genes (SLC31A1 and LIAS) based on LightGBM was constructed, and its good performance in the specific recognition of AF was validated. Cuproptosis may be regulated by HOXA9 and TET1 in AF. Moreover, cuproptosis and immunity may orchestrate the pathogenesis of AF. This comprehensive analysis provides the possibility to improve the diagnosis for patients with AF and provides a theoretical base for upcoming research on the associations between immunity and cuproptosis-related genes in AF.

### Electronic supplementary material

Below is the link to the electronic supplementary material.

Supplementary Material 1

## Data Availability

The datasets supporting the findings of this study are open-access from GEO (https://www.ncbi.nlm.nih.gov/geo/).

## Abbreviations

AF: Atrial fibrillation
ATP7A: ATPase copper transporting Alpha
AUC: Area under the curve
BP: Biological progress
DLST: Dihydrolipoamide s-succinyltransferase
EL: Ensemble learning
GCSH: Glycine cleavage system protein H
GEO: Gene Expression Omnibus
GO: Gene ontology
GSEA: Gene set enrichment analysis
HOXA9: Homeobox A9
KEGG: Kyoto Encyclopedia of Genes and Genomes
LIAS: Lipoic acid synthetase
LightGBM: Light Gradient Boosting Machine
ML: Machine learning
OAC: Oral anticoagulation
PCA: Principal component analysis
PR: precision-recall
RF: Random Forests
ROC: Receiver operator characteristic
SLC31A1: Solute carrier family 31 member 1
SR: Sinus rhythm
TET1: Tet methylcytosine dioxygenase 1
TF: Transcription factor
WD: Wilson’s disease
XGBoost: eXtreme Gradient Boosting
